# When donor funding leaves: an interrupted time-series analysis of the impact of integrating direct HIV care and treatment into public health services in a region of Johannesburg

**DOI:** 10.1186/s12962-019-0192-5

**Published:** 2019-10-25

**Authors:** Naomi Lince-Deroche, Rahma Leuner, Gesine Meyer-Rath, Yogan Pillay, Lawrence Long

**Affiliations:** 1 Health Economics and Epidemiology Research Office, Department of Internal Medicine, School of Clinical Medicine, Faculty of Health Sciences, University of the Witwatersrand, 39 Empire Rd, Parktown, Johannesburg, 2194 South Africa; 2 Department of Global Health, School of Public Health, Boston University, Boston, USA; 3 South African National Department of Health, Pretoria, South Africa

**Keywords:** Integration, Donor funding, Primary healthcare, HIV, South Africa, National health databases

## Abstract

**Background:**

Early in South Africa’s HIV response, donor-funded organizations directly provided HIV treatment through Comprehensive HIV Care, Management and Treatment sites (CCMTs), using their own and government staff. From 2012 to 2014 the donor-funded CCMT model was phased out, leaving nurses in South Africa’s public clinics responsible for delivery of antiretroviral treatment (ART) services. We aimed to examine the impact on resources, staff workloads, and service delivery throughout this period of integration of HIV treatment into primary health clinics.

**Methods:**

We conducted an Interrupted Time-Series Analysis (ITSA) using data from three public clinics, including one former CCMT site, in one administrative region of Johannesburg. The ITSA was complemented by visual inspection of the data in Excel. We compared trends in expenditure, clinical staffing levels, patient headcounts, and services rendered at the clinics during four periods: pre-CCMT (2004–2007), CCMT operational (2007–2012), CCMT closure (2012–2014), and post-CCMT (2014–2016). Data were drawn from the country’s District Health Information System, a national HIV treatment database, local budget and expenditure reports, National Health Laboratory Service charge records, and staff records.

**Results:**

Closure of the CCMT differentially impacted the study clinics. As expected, ART services decreased at Clinic 1, where the CCMT was co-located, and increased at Clinics 2 and 3 possibly reflecting redistribution of patients. Despite a reduction in patient headcounts post-CCMT, Clinic 1 experienced a decrease in staff and a large increase in patients seen per clinical staff member per month. In contrast, Clinics 2 and 3 increased or maintained stable workforces, and staff workloads post closure were similar to pre-closure levels. Other primary care services—contraception and immunisations—seemed largely unaffected at Clinics 1 and 2. At Clinic 3, service delivery reduced, but this was accompanied by lowered patient headcounts generally, likely due to clinic renovations.

**Conclusions:**

In this study, integration of HIV treatment into primary healthcare services did not result in large-scale reductions in overall service delivery. One facility did experience increased staff workloads, but we were unable to assess service quality. To mitigate potential problems, monitoring systems should be introduced in advance and acknowledge the disparate and decentralised management of various data sources.

## Background

One of five people living with HIV globally live in South Africa [1, 2]. The United States President’s Emergency Plan for AIDS Relief (PEPFAR) was established in 2003 with the aim of helping to combat the global HIV/AIDS epidemic [3]. PEPFAR’s role in South Africa has varied over time. PEPFAR began supporting HIV care and treatment in South Africa in 2003. However, South Africa differs significantly from other PEPFAR-supported countries in that, barring provision by a small number of private or NGO providers, the costs of antiretroviral drugs have always been fully borne by the South African government. Further, over time, the vast majority of additional funding required for the country’s HIV response has shifted to domestic sources [4]. In financial year 2014/15, the South African government was responsible for funding 78% of its domestic HIV response [5].

In its first phase, from 2003 to 2008, PEPFAR was an emergency plan for dealing with AIDS-related morbidity and mortality globally. In South Africa, implementing partners were funded to assist government in providing HIV services, including providing antiretroviral therapy (ART), monitoring of disease progression, and care for opportunistic infections. Treatment was provided directly to patients via Comprehensive Care, Management and Treatment facilities, or CCMTs. PEPFAR’s second phase, from 2009 to 2012, emphasized increasingly collaborative partnerships with local governments, and most recently, in PEPFAR phase three, responsibility for leadership and service delivery has fully shifted from partner organizations to provincial governments [6].

As in other PEPFAR-supported countries, during PEPFAR phases one and two, implementing partners in South Africa received support to provide direct HIV care and treatment via CCMTs. These were specialist HIV treatment clinics operating on the grounds of a subset of public healthcare facilities across the country, staffed by both government-employed and implementing partner staff. From 2012 to 2014, CCMTs were phased out, and responsibility for HIV treatment was transferred to public facilities run by the South African government. In parallel with these funding shifts, South Africa in 2010 and again in 2011 adopted new treatment guidelines in line with World Health Organization (WHO) recommendations [7–9]. The country also phased in nurse-initiated and managed ART (NIMART) (as opposed to exclusively doctor initiated/managed care) in public facilities [10] to address increasing demand for HIV treatment services. In this study, we examined changes in financial and human resources, staff workloads, and service volumes during and after the transition of responsibility for HIV treatment from a CCMT to three adjacent public sector facilities in one region of Johannesburg, South Africa. Our aim was to illustrate the short term impact of the integration of HIV treatment services into public sector primary healthcare facilities, focusing on available resources as well as the delivery of two other public health services, including specifically contraceptive and child immunisation services.

## Methods

### Study location

There are seven administrative regions in the City of Johannesburg in Gauteng Province, South Africa. Each municipal region is responsible for the delivery of primary and environmental healthcare services to its inhabitants [11]. We used purposive sampling to choose three public healthcare clinics within one municipal region of the City. All were under the jurisdiction of the City (as opposed to the Province). Clinic 1 was a relatively large clinic, which previously housed a CCMT on its grounds, and was located in an under-served, peri-urban, densely populated area. Clinic 2, which was smaller than Clinic 1, was located about 2 km away and served the same population. Clinic 3—also a small clinic—was located about 12 km away from Clinic 1 in a more developed urban area which was home to most of the approximately 250,000 residents of the region [11].

### Data sources

We conducted a retrospective evaluation using healthcare utilisation and expenditure data from several sources: South Africa’s District Health Information System (DHIS), Tier.net data, clinic- and province-level expenditure reports, and staff records supplied by the municipal regional health authority.

The DHIS is South Africa’s official system for tracking service delivery metrics in the public health sector. It is used for recording the number of routine health services rendered by public health facilities on a monthly basis. Data collected at the primary healthcare level include the number of individuals who receive HIV testing, ART, tuberculosis testing and treatment, child health, immunisations, contraception, and other women’s health services. The DHIS also contains patient headcounts, which represent the total number of patients who have visited a clinic in a given month. The source documentation for the DHIS is paper registers maintained by staff in clinics, and although the data are checked at various stages during compilation, they are prone to errors [12, 13]. Nonetheless, we obtained monthly DHIS data for the period of 2004 to 2016 for this analysis from the City of Johannesburg.

For presentation of contraceptive data from the DHIS, we summed the number of visits reported for delivery of subdermal contraceptive implants, intra-uterine contraceptive devices (IUCDs), medroxyprogsterone injections, norethisterone enanthate injections, and oral contraceptive pills. Condoms visits were excluded to avoid double counting as they were often distributed at the same time as several other services. We also obtained immunisation data from the DHIS. These data represent the number of under-1-year-olds reported to have been fully immunised at each clinic—meaning they presented for their last under-one vaccination and were found to have had all previously required doses as well.

Tier.net is an electronic database that captures patient-level information regarding ART services rendered, including visit dates, viral load results, and drugs administered. As with the DHIS, the original source of this information is paper-based. Tier.net was implemented in 2014. At that time, older data from a prior electronic system (Therapy-Edge) were migrated into Tier.net in the clinics under study. Thus we were able to obtain monthly Tier.net data for the period of 2004 to 2016 from the study clinics.

We also obtained annual staffing data from the municipal regional authority and the implementing partner that ran the CCMT at Clinic 1. Staff workloads per annum were estimated by dividing the number of clinical staff members (i.e. doctors, nurses, dieticians, etc.) by the patient headcounts derived from the DHIS.

We obtained monthly public sector laboratory cost data from the National Health Laboratory Service (NHLS), the sole provider of laboratory services for the public health sector. These data are presented as NHLS *charges* to the state expressed in 2016 ZAR. Using charges is not ideal if they do not reflect actual costs or expenditure. However, as the analysis is meant to reflect the perspective of the health care system, i.e. the clinics where care was provided, then charges do in fact reflect the resource requirements for providing care.

Finally, we were unable to obtain pharmacy expenditure data (i.e. medication costs) for this analysis. Also, while we obtained monthly clinic-level expenditure data from South Africa’s Basic Accounting System for 2009 onward, these data were found to be “lumpy” in nature—i.e. representing bulk expenditure at discreet time points, rather than real time expenditure—and so were deemed inappropriate for this analysis.

### Data management and analysis

All data were first manipulated and reviewed for inconsistencies in Excel (2013, Microsoft Corporation). This involved producing simple graphic representations of the data to view changes over time. Discrepancies, if found, were discussed with the source of the data and corrected.

We then compiled final datasets in Excel and exported the data to Stata (StataCorp. 2015. Stata Statistical Software: Release 14. College Station, TX: StataCorp LP). Using recommended methods for Interrupted Time-Series Analysis (ITSA) [14] and visual analysis [15], we compared resource data at all three study clinics across four comparison periods: (1) before establishment of the CCMT (2004–2007), (2) CCMT operational (2007–2012), (3) CCMT close-out occurring (2012–2014), (4) CCMT closed (2014–2016). We used ITSA where monthly data were available, allowing for the required minimum of 10 observations per period [11]. Where annual data were available, we used visual inspection only due to the limited number of observations.

In an ITSA, outcome variables are compared over multiple time periods before and after the introduction of one or more interventions that are expected to interrupt the variable’s *level* and/or *trend* [14]. An ITSA model assumes linear trends over time, and cannot be used to determine whether fluctuations in the data represent pre-emptive reactions to forthcoming interventions or delayed responses to past interventions. These problems have been mitigated in this analysis, following recommended practice [15], by conducting the ITSA in parallel with visual inspection of the data. Visual analysis should involve examination of within- and between-phase data patterns. Within-phase analysis involves an examination of levels, trends, and variability. Between-phase analysis involves the consideration of immediacy of effect, overlap, and consistency of effect [15].

For this analysis, the ITSA assumed the following functional form:

$$\begin{aligned} {\text{Y}}_{\text{t}} & = \upbeta_{0} + \upbeta_{1} {\text{T}}_{\text{t}} + \upbeta_{2} {\text{X}}_{2007} + \upbeta_{3} {\text{X}}_{2007} {\text{T}}_{2007} \hfill \\ & \quad + \upbeta_{4} {\text{X}}_{2012} + \upbeta_{5} {\text{X}}_{2012} {\text{T}}_{2012} + \upbeta_{6} {\text{X}}_{2014} \hfill \\ & \quad + \upbeta_{7} {\text{X}}_{2014} {\text{T}}_{2014} + \upepsilon_{\text{t}} \hfill \\ \end{aligned}$$

where Y_t_ is the aggregate outcome variable measured at each equally spaced time point t, T_t_ is the time since the start of the study, X_t_ are dummy variables representing the intervention, and X_t_T_t_ are interaction terms. β_0_ is the intercept, or starting level of the outcome variable at the beginning of period 1 (i.e. pre CCMT), and β_1_ is the slope of the outcome variable throughout period 1. β_2_, β_4_, and β_6_ represent immediate treatment effects [14]. These represent comparisons of the level of the outcome at the start of each period. For example, β_2_ represents a comparison of the intercept (i.e. the start of period 1) to the outcome at the start of period 2 when the CCMT became operational. Likewise, β_4_ represents the change in the outcome comparing the start of period 2 (i.e. CCMT operational) to the start of period 3 (i.e. CCMT close-out), and β_6_ represents the change in the outcome comparing the start of period 3 (i.e. CCMT close-out) to the start of period 4 (i.e. CCMT close-out completed). β_3_, β_5_, and β_7_ represent treatment effects over time [14]. Specifically, β_3_ is the change in slope from period 1 to period 2; β_5_ represents the change in the slope between period 2 and period 3; and β_7_ represents the change in slope between periods 3 and 4. ℇ_t_ represents an error term.

The study received prior approval from the Human Research Ethics Committee at the University of the Witwatersrand and from the City of Johannesburg, the municipal regional authority, and the study facilities.

## Results

### Timeline of ART provision and integration at the three study clinics

**Fig. 1:**
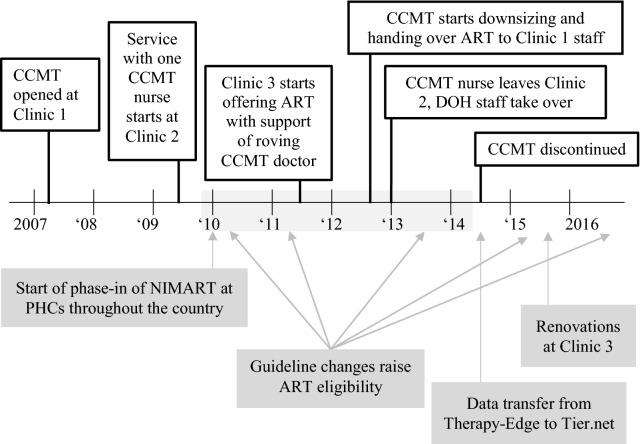
Timeline of events during period of integration of HIV treatment into public clinic service offering. *CCMT* comprehensive HIV care, management and treatment site, a “container clinic” supported through donor funding and located on the grounds of public sector clinic. The CCMT offered antiretroviral treatment directly to patients. *DOH* Department of Health, i.e. government-employed staff, *PHCs* primary healthcare clinics, *ART* antiretroviral treatment, *NIMART* nurse-initiated and managed ART

Figure 1 presents a timeline of antiretroviral service provision at the three study clinics. The CCMT, housed in a shipping container, opened on the grounds of Clinic 1 in 2007 and began offering HIV testing and treatment. Government-employed staff offered HIV testing only. When the CCMT started operating, staff at Clinics 1, 2, and 3 were able to refer HIV-positive patients to the CCMT at Clinic 1 for treatment. This referral arrangement changed in 2009, when the increase in patient load necessitated that Clinic 2 be initiated as a satellite facility for the CCMT at Clinic 1. At that time, a single, donor-funded nurse at Clinic 2 began offering ART services. As at Clinic 1, the government-employed staff at Clinic 2 continued offering HIV testing, but no ART services. Clinic 3 never had donor-funded staff providing HIV treatment at the clinic. However, a roving doctor employed by the implementing partner was responsible for training government-employed staff at a number of clinics in the area, and Clinic 3 started offering ART to its patients—provided by its own nursing staff—by mid-2011.

Downsizing of the CCMT started in late 2012. This involved training and starting the transfer of responsibility for providing ART to the government-employed staff at Clinic 1. The satellite nurse at Clinic 2 also left in late 2012, leaving the clinic staff there responsible for ART. By mid-2014, the CCMT had largely closed. The government-employed staff at Clinic 1 were then responsible for ART provision for their patients (as were the staff at Clinics 2 and 3). Also depicted in Fig. 1 are other changes in the region that occurred over the period of observation.

### Patient headcounts

**Fig. 2:**
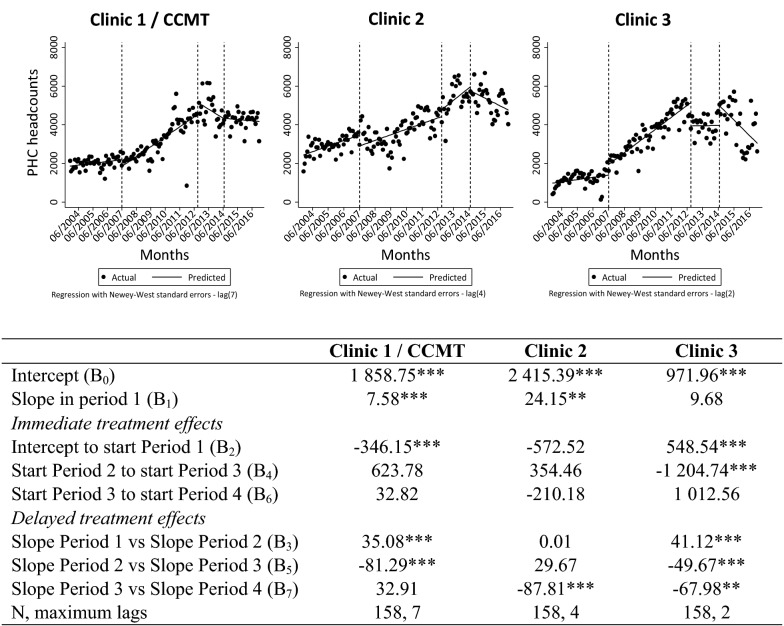
ITSA of patient headcount data throughout transition of HIV treatment to government-employed staff. *p < 0.1, **p < 0.05, ***p < 0.01. *ART* antiretroviral treatment, *PHC* primary healthcare service, *ITSA* interrupted time-series analysis, *CCMT* comprehensive HIV care, management and treatment site. Vertical lines represent the start/stop of each period in the visual representation. Period 1 = pre CCMT, no ART offered; Period 2 = CCMT operational at Clinic 1, ART offered by CCMT staff; Period 3 = CCMT close-out happening, ART transition to government-employed staff in progress; Period 4 = CCMT close-out complete, government-employed staff provide all ART

Patient headcount data reflects the total number of patients seen in a clinic for any condition. Visual examination of the patterns in Fig. 2 shows that all three clinics experienced an increase in patient headcounts during the CCMT’s operational phase. Based on the ITSA results in Fig. 2, the headcount level at Clinic 1 decreased significantly (p < 0.01) prior to opening of the CCMT (in period 1) (comparing the intercept to the start of the CCMT’s operational period). Then the slope in patient headcounts changed significantly (p < 0.01), from a decreasing trend to an increasing trend when comparing period 1 (pre-CCMT) to period 2 (CCMT operational). The trend changed significantly again (p < 0.01) to a decrease in patient headcounts during period 3 (CCMT close-out). At Clinic 2, headcounts increased during CCMT close-out (though not significantly so) possibly due to absorption of patients from Clinic 1. Interestingly, the slope in patient headcounts changed significantly (p < 0.01) from an increasing trend during period 3 (CCMT close-out) to a decline in period 4 (CCMT closed). However, visually, headcounts after the closure remained higher than pre-closure levels. Clinic 3 experienced patient headcount fluctuations over time including a significant change in slope (from increasing to a “flat,” or neutral, trend, p < 0.01) when comparing period 2 (CCMT operational) and period 3 (CCMT close-out). In period 4 (post CCMT closure), the absolute number of patients was higher than in period 3 (though not significantly so), but the change in trend from period 3 to period 4 was again significant (p < 0.05) (from neutral to a decline), likely due to clinic renovations in 2015.

### Staffing and staff workload

**Fig. 3:**
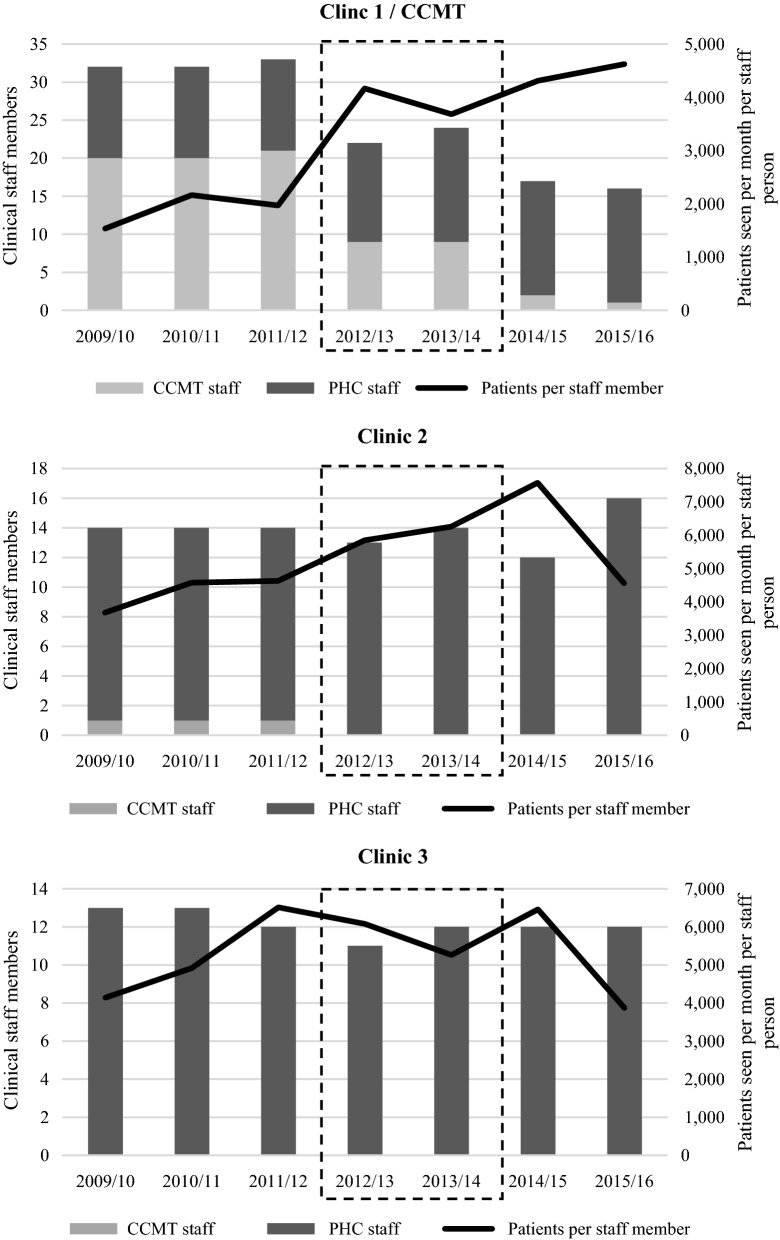
Clinical staffing and total monthly patients per clinical staff member before, during, and after CCMT*. *CCMT* comprehensive HIV care, management and treatment site. These staff were supported by donor funding. *PHC* primary healthcare clinic staff, employed through public funds by the Department of Health. Dashed box represents CCMT close-out period (i.e. mid 2012–mid 2014). *Data represent financial years 2009–2015

Figure 3 presents the total number of clinical staff members at each clinic, including both CCMT staff and staff members employed by the municipal regional authority (i.e. the Department of Health) over time. Excluded from the clinical staff count are administrative assistants, cleaners, data managers, etc. Unfortunately, staffing data were not available for period 1, i.e. pre-CCMT, and thus the timeline begins when the CCMT was operational at Clinic 1. The number of total staff at Clinic 1 dropped from 33 in 2011/12, when the CCMT was operational, to 22 in 2012/13, when CCMT closure began, and dropped again to 17 in 2014/15 when CCMT closure was complete. The decrease reflected the loss of CCMT staff at the clinic. The Department of Health employed three new staff members during and post CCMT closure (in 2012 and 2014) in an attempt to compensate. However, despite the extra staff, workloads for clinical staff members at Clinic 1 increased dramatically after CCMT closure. As shown in Fig. 3, the number of patient visits per clinical staff member per month reportedly increased from 1969 in 2011/12 to 4313 in 2014/15. These numbers suggest an average of just over 2 min of face-to-face contact per patient. We learned from clinic staff that the headcounts represent all individuals who attend the clinic, including for group counselling or to accompany a friend or relative, so the actual amount of time per patient may be higher. Regardless, interestingly, clinical staff at Clinics 2 and 3 were seeing similar numbers of patients post CCMT closure. At Clinic 2 the average number of patients seen per clinical staff person peaked at over 7000, but was brought down to roughly 4500 post closure. At Clinic 3, the number was slightly under 4000 per clinical staff member per month.

At Clinic 2, the nurse affiliated with the CCMT left when CCMT closure began in 2012/13; however, that gap was filled by Department of Health staff in 2013/14. There were other staff losses in 2014/15, post-CCMT; however, the Department was able to hire additional staff the following year. Although staff workloads (defined as patients seen per clinical staff member per month) increased at Clinic 2 during the CCMT’s closure—possibly due to absorption of CCMT patients—workloads later decreased dramatically due to the additional staff. Staffing levels and workload at Clinic 3 fluctuated but remained relatively stable over time, with the exception of a drop in workload when the clinic was undergoing renovations in 2015/16.

### ART visits

**Fig. 4:**
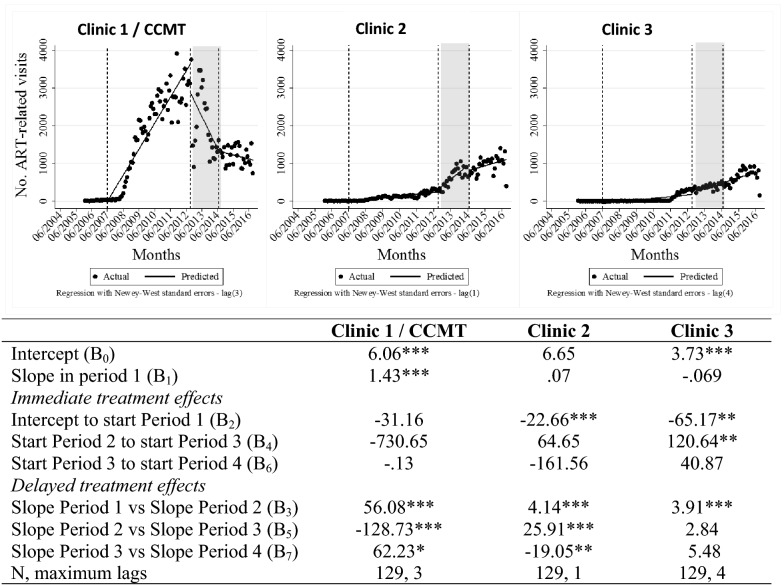
ITSA of ART visit data throughout transition of HIV treatment to government-employed staff. *p < 0.1, **p < 0.05, ***p < 0.01. *ART* antiretroviral treatment, *ITSA* interrupted time-series analysis, *CCMT* comprehensive HIV care, management and treatment site. Vertical lines represent the start/stop of each period in the visual representation. Period 1 = pre CCMT, no ART offered; Period 2 = CCMT operational at Clinic 1, ART offered by CCMT staff; Period 3 = CCMT close-out happening, ART transition to government-employed staff in progress; Period 4 = CCMT close-out complete, government-employed staff provide all ART

Figure 4 depicts the number of ART initiation or medication pick-up visits made by patients at each study clinic over time and ITSA results comparing absolute numbers and trends across the periods. As expected, after experiencing a steep increase in ART visits when the CCMT was operational, Clinic 1 experienced a statistically significant (p < 0.01) change in trend when the CCMT was closing and distributing ART patients to other clinics in the region. In fact, the decline in ART visits at Clinic 1 appears to have started just prior to the closing of the CCMT, potentially due to other clinics in the region initiating NIMART before mid-2012. Clinic 2 began offering ART through its one CCMT nurse in 2009; Clinic 3 began offering ART as part of NIMART services in 2011/2012. Based on visual inspection, as expected, both clinics experienced increases in the absolute number of ART visits during and post CCMT closure. The ITSA revealed that the increase in visits at Clinic 3 was significant (p < 0.05) when comparing the CCMT operational and closure periods. At Clinic 2, ART visits had been increasing during the CCMT operational period, and the rate of increase (i.e. the slope) increased significantly (p < 0.01) during closure. The decline in the number of visits at Clinic 1 appears greater than the combined increases at Clinics 2 and 3; however, it is important to remember that there were other clinics in the region that were also integrating ART services into their primary care offering at this time. Unfortunately, monitoring ART services at all clinics in the region was outside the scope of this analysis.

### NHLS charges

**Fig. 5:**
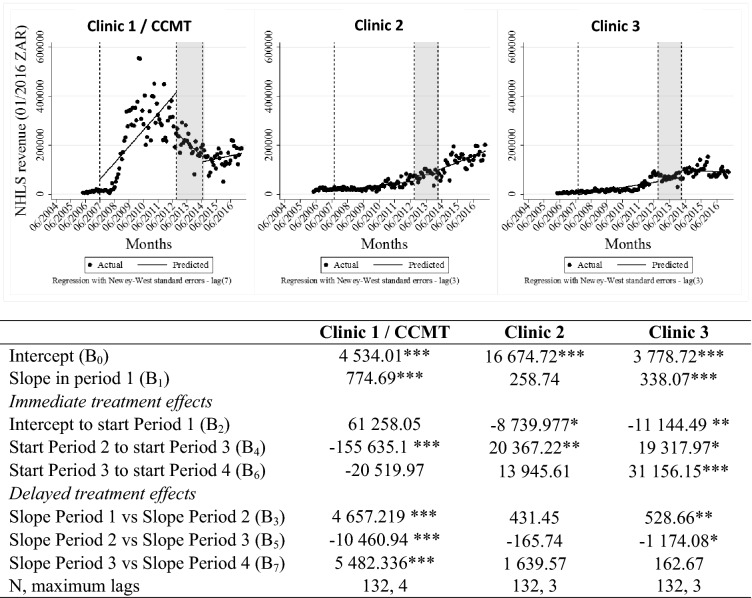
ITSA of national laboratory revenue data throughout transition of HIV treatment to government-employed staff. *p < 0.1, **p < 0.05, ***p < 0.01. *ART* antiretroviral treatment, *ITSA* interrupted time-series analysis, *CCMT* comprehensive HIV care, management and treatment site. Vertical lines represent the start/stop of each period in the visual representation. Period 1 = pre CCMT, no ART offered; Period 2 = CCMT operational at Clinic 1, ART offered by CCMT staff; Period 3 = CCMT close-out happening, ART transition to government-employed staff in progress; Period 4 = CCMT close-out complete, government-employed staff provide all ART

Figure 5 depicts changes in NHLS charges (expressed in 2016 South African Rand) at the three study clinics. These data represent charges for any laboratory investigation. NHLS charges for Clinic 1 declined when comparing the CCMT operational period to the closure period, both in terms of the absolute value (p < 0.01) and the change in trend (p < 0.01). At Clinics 2 and 3 NHLS charges increased significantly (p < 0.05 and p < 0.1 respectively) when comparing the CCMT operational period to the closure period. Overall, changes in the NHLS charges seem to have been largely driven by the costs of monitoring HIV-positive individuals on treatment, as the trends closely mirror the trends in ART visits at the clinics. However, the two datasets do differ in that: (1) the decline in NHLS charges at Clinic 1 preceded the decline in ART visits, and (2) there was an increase in NHLS charges at Clinic 1 from late 2015 onward, which were not seen in the ART visit data. Both differences may be explained in part by changes in recommendations for lab-based monitoring or price changes at the NHLS.

### Delivery of other primary healthcare services

**Fig. 6:**
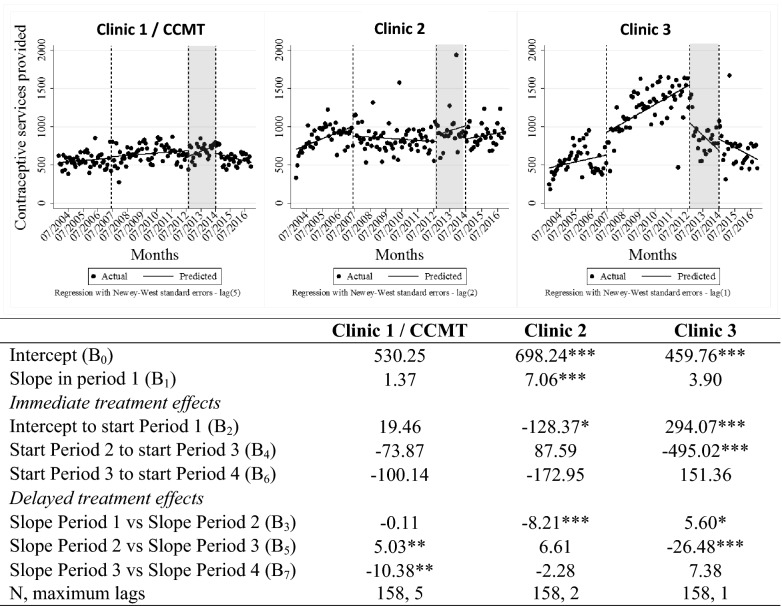
ITSA of contraceptive service delivery data throughout transition of HIV treatment to government-employed staff. *p < 0.1, **p < 0.05, ***p < 0.01. *ART* antiretroviral treatment, *ITSA* interrupted time-series analysis, *CCMT* comprehensive HIV care, management and treatment site. Vertical lines represent the start/stop of each period in the visual representation. Period 1 = pre CCMT, no ART offered; Period 2 = CCMT operational at Clinic 1, ART offered by CCMT staff; Period 3 = CCMT close-out happening, ART transition to government-employed staff in progress; Period 4 = CCMT close-out complete, government-employed staff provide all ART

**Fig. 7:**
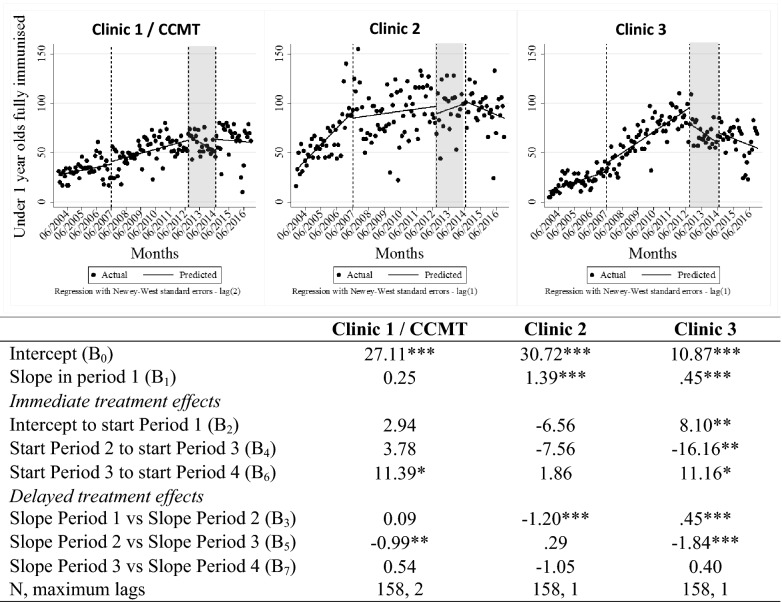
ITSA of immunisation service delivery data throughout transition of HIV treatment to government-employed staff. *p < 0.1, **p < 0.05, ***p < 0.01. *ART* antiretroviral treatment; *ITSA* interrupted time-series analysis, *CCMT* comprehensive HIV care, management and treatment site. Vertical lines represent the start/stop of each period in the visual representation. Period 1 = pre CCMT, no ART offered; Period 2 = CCMT operational at Clinic 1, ART offered by CCMT staff; Period 3 = CCMT close-out happening, ART transition to government-employed staff in progress; Period 4 = CCMT close-out complete, government-employed staff provide all ART

Figures 6 and 7 illustrate delivery of contraceptive and immunisation services at the three study clinics throughout the period of interest. Visual inspection of the contraceptive data presented in Fig. 6 shows that provision of contraceptive services was relatively stable over time at both Clinics 1 and 2. According to the ITSA, at Clinic 1 there was a small but significant (p < 0.05) change in the slope (to a sharper incline) when comparing the CCMT operational period to the closure period. Also at Clinic 1, the slope, or tend, changed again (from increasing to decreasing) (p < 0.05) post CCMT closure. Fluctuations in service provision at Clinic 2 were non-significant (post the CCMT operational period). Clinic 3 also experienced fluctuations over time. The change in trend from period 2 (the CCMT operational phase), where Clinic 3’s contraceptive services were increasing, to period 3 (close-out), where services declined, was significant (p 0.01). The reason for the decline in period 3 is unclear.

Figure 7 provides data representing the number of under-1-year-olds reported to have been fully immunised at each clinic. Visual inspection suggests a steady increase in immunisations at Clinic 1 until the beginning of 2011. However, the ITSA shows that the increase from pre-CCMT to the CCMT’s operational phase was not significant. That said, the trend did change from increasing to decreasing (p < 0.05) during CCMT closure. At Clinic 2, there were no statistically significant changes to immunisation services–in level or trend–during or after CCMT close-out. At Clinic 3, the trends for immunisations increased (p < 0.01) during the CCMT’s operational period (period 2) and then changed to a decrease (p < 0.01) during closure (period 3).

## Discussion

This analysis sheds light on the impact of integrating HIV treatment services into routine primary healthcare service delivery at three clinics in a region of Johannesburg. Specifically, the data provide insight regarding the impact on service delivery, staffing, and staff workloads.

Although we were unable to track the movement of individual patients, patient headcount data supported expectations of ART patients being distributed from the CCMT to primary healthcare facilities during the period of transition. At Clinic 2, which was physically the closest to Clinic 1 where the CCMT was located, the overall patient headcount increased as ART visits increased. At Clinic 3, the picture was less clear. ART visits increased, but the patient headcount trend stabilized during the transition phase. This was despite decreases in both contraception and immunisation services at Clinic 3 during the CCMT close-out period. Immunisation services later recovered to levels seen during the CCMT’s operational phase. Contraceptive services continued to decline, but this may have been related to other factors, including the clinic’s renovations.

Nonetheless, our conclusion is that introduction of ART services at Clinic 3 did not result in large-scale reductions in the level of service provision for contraceptive and immunisation services, which may serve as a proxy for other primary healthcare services. This was true at Clinics 1 and 2 as well. At Clinic 1, ART visits declined as patients were re-distributed to other sites, possibly clinics 2 and 3, but possibly also other facilities. Contraceptive and immunisation services decreased slightly—post and during the closure respectively. This may have been the result of reduced demand as patient headcounts decreased during the closure period. At Clinic 2, contraceptive and immunisation services remained stable despite the increase in ART patient visits.

Interestingly, for most of the observation period, fewer contraceptive services were provided at Clinic 1 than at either of the other two study clinics, which were smaller in size. Also, the country launched the contraceptive subdermal implant in 2013/14, but it does not appear to have had a major impact in terms of overall service volume at any clinic.

When considering the stability of patient service statistics over time, one must consider staffing and staff workload issues. This analysis shows that Clinic 1 lost many staff members—who were assigned to the CCMT and supported by donor funding—when the CCMT closed. They were not absorbed into the Department of Health’s complement at Clinic 1. As a result, the monthly patient workload per clinical staff member at Clinic 1 increased drastically when the CCMT closed. Some new staff were hired, but overall the workload remained higher post closure than pre-closure. Conversely, Clinics 2 and 3 managed to hire or maintain staff and reduce their patient workloads over time to roughly pre-CCMT levels. It is unclear why Clinics 2 and 3 were able to maintain stable staff workloads, i.e. whether they had more autonomy or money for new staff. All three clinics were under the administration of the same municipal regional authority, and differences in available resources were unlikely. It is also unclear whether the municipal regional authority considers an ideal provider to patient ratio when hiring or redeploying staff. When comparing the three clinics post closure, clinical staff workloads were similar (at between 4000 and 5000 patients seen per month per clinical staff person). It is possible that staff at Clinic 1 were viewed as serving too few patients per month prior to CCMT closure, and post closure, staffing was adjusted to bring monthly workloads in line with other clinics. Possibly supporting this theory, the monthly provider to patient headcount ratio rose due to loss of staff at Clinic 2 just after CCMT closure, and the municipal regional authority hired staff, bringing the ratio back in line with the ratios observed post closure at the other clinics.

There are limitations to this work. First and foremost, the data were derived from several different sources and reflect service delivery at just three of many clinics in the City of Johannesburg. The data are also imperfect. They represent collated information drawn from paper-based registers and other sources that are known to be inaccurate at times. For example, it is unclear how well coordinated patient headcount tracking was between the CCMT’s implementing partner and the Department of Health during the CCMT’s operational phase. The DHIS headcount data may have underestimated patient headcounts if the CCMT data was not always incorporated into the clinics’ registers. This would result in an underestimate of headcounts and staff workload during the CCMT’s operational phase in this analysis. There was also no way to determine whether patients moved from one study clinic to another study clinic (as compared to shifting to/from non-study clinics), and finally, it is important to note that, for immunisation services, we presented data for under-1-year-olds who had received all required vaccinations, including the last one which prompted documentation in the DHIS data. Prior vaccinations could have been received at other facilities.

Despite these potential weaknesses in the data, they are the best available information, and do help to shed light on changes to service delivery patterns within facilities over time. In future analyses, sensitivity analyses that vary the headcount data—to account for possible inaccuracies—could be helpful. Future analyses could also benefit from including individual vaccinations which may be more facility- or time-dependent.

From an analytic standpoint, it bears mentioning that ITSA and visual inspection are both imperfect methods for evaluating single-case data. The ITSA model assumes linear trends over time, which may not always be accurate, and cannot be used to determine whether there was a pre-emptive reaction to an expected outcome or a delayed response to it [15]. ITSA can benefit from including a control—in this case, for example, a facility not affected by the changes occurring at the intervention facilities. However, as the funding and service delivery changes at the intervention facilities were occurring nationwide, this was not possible. On the other hand, visual analysis can be subjective and cannot be used to ascertain the exact magnitudes of effects [15]. These problems have been mitigated in this analysis, following recommended practice, by conducting the ITSA in parallel with visual inspection of the data [15].

Finally, there were significant changes to HIV management in South Africa’s public sector throughout the period of observation, including the introduction of NIMART services, the shifting of ART thresholds, and the transfer of ART record keeping. The ITSA model estimates are also not controlled for covariates, implying that background population characteristics (e.g. large-scale clinic catchment area growth or reduction) do not change during the study period. We attempted to mitigate this risk through conducting interviews on regional service delivery with key stakeholders (these have been published separately). They did not report major demographic changes in the study communities during the period of observation; however, recall bias may have still contributed to undocumented background changes.

Other studies evaluating the introduction of HIV treatment services into primary healthcare in South Africa, of which there are few, report poor preparation for the transition. Based on interviews with 75 people directly involved in the transition as well as media reports, Kavanagh [16] found that patients experienced long waits, being turned away, severe stigma, poorly prepared staff, and stock-outs of ART and tuberculosis drugs at many clinics [16]. Certain clinics also experienced disrupted care and increased loss-to-follow up. Cloete et al. [17] showed that 16% of ART patients who previously obtained care at a PEPFAR site were lost-to-follow-up in that they did not visit a public clinic in a 4-month period following the transition [17]. Kavanagh [16] also reported on the inability of government to absorb PEPFAR-funded staff such as doctors, nurses, adherence counsellors, and data administrators into the public sector after the transition, leading to government-employed staff being overwhelmed with work [17].

Our findings are more nuanced, albeit less in-depth. Although Clinic 1 staff did see a significant change in workload, the same did not occur in the longer term at Clinics 2 and 3, despite some increases in patient headcounts. Our analysis cannot speak to service quality or waiting times at any of the facilities. However, having a greater patient workload may not necessarily equate to worse service provision in all cases. The withdrawal of the CCMT happened alongside service integration, so presumably at Clinic 1 post-CCMT patients could receive their HIV treatment and other primary healthcare services in one consultation or with less movement within the clinic and clinic grounds. At Clinics 2 and 3, patients could receive their HIV treatment closer to home.

## Conclusion

Our data suggest that—against a backdrop of increasing access to HIV treatment services generally—integration of HIV treatment services into the primary healthcare service offering at three public sector clinics in Johannesburg did not result in large-scale reductions in overall service delivery. However, staff workloads were dramatically increased in at least one case, and it is unclear what the impact was on service quality. To mitigate potential problems in other settings, monitoring systems should be prepared in advance and acknowledge the often disparate and decentralised management of various data sources. To allow for successful community-based service delivery, clinical and clerical healthcare workers should be capacitated to plan for changes in workload, and to contribute to and review data collected for quality and service management purposes.

## Data Availability

The data that support the findings of this study are available from City of Johannesburg, the South African National Department of Health, and the National Health Laboratory Service. Restrictions apply to the availability of these data, which were used under license for the current study, and so are not publicly available. Data are however available upon reasonable request to the authors with permission of the guardians of this data and a local ethics committee, which can be reached at hrec-medical.researchoffice@wits.ac.za.
